# The Biology of Demodecid Mites (Trombidiformes: Demodecidae) and Their Parasitism in the Eurasian Beaver *Castor fiber* Linnaeus, 1758, with a Description of a New Species †

**DOI:** 10.3390/ani15142136

**Published:** 2025-07-18

**Authors:** Leszek Rolbiecki, Joanna N. Izdebska, Joanna Dzido, Sławomira Fryderyk

**Affiliations:** Department of Invertebrate Zoology and Parasitology, Faculty of Biology, University of Gdańsk, Wita Stwosza 59, 80-308 Gdańsk, Poland; joanna.izdebska@ug.edu.pl (J.N.I.); joanna.dzido@ug.edu.pl (J.D.); slawomira.fryderyk@ug.edu.pl (S.F.)

**Keywords:** *Castor fiber*, co-occurring parasites, demodecids topography, *Demodex castoris*, *Demodex ovaportans* sp. nov., host–parasite interaction, skin mites

## Abstract

The largest Eurasian rodent, the Eurasian beaver *Castor fiber*, is known for its amphibious lifestyle that allows it to adapt its environment to its needs. Due to its lifestyle and evolutionary history, the beaver is characterized by a distinct, unique parasitofauna. In this context, the occurrence of mites from the Demodecidae family in the Eurasian beaver was investigated. The topography of the *Demodex castoris* was analyzed: it was previously known from a single record from a single skin location of this host. The mite was found in large numbers in various locations in the hairy skin, including the head, trunk, and limbs. In addition, a new species associated with hairless skin, mainly around the mouth, was discovered and described: *Demodex ovaportans* sp. nov. The females of this species carry the egg on the dorsal side of the podosoma, which may be a form of care and a previously unknown reproductive strategy in Demodecidae. Our findings confirm that a host-specific demodecid mite species associated with the hairy skin of the entire body is a universal model in mammals. They also emphasize the uniqueness of the beaver parasitofauna, as evidenced by the host specificity and the different biology of the demodecids described in it.

## Simple Summary

Two species of parasitic mites from the family Demodecidae were identified in the skin of the Eurasian beaver *Castor fiber*, each occupying distinct microhabitats. Also, representatives of a new species, *Demodex ovaportans* sp. nov., associated with hairless skin, mainly around the mouth, were found. In contrast, *Demodex castoris*, previously known only from a single record in the nasal area, was detected across the entire hairy skin of the body. These findings confirm a widely observed universal pattern of parasitism in demodecid mites, where the mammal host is inhabited by a host-specific species associated with the hairy skin region, which are accompanied by additional mite species occupying separate microhabitats. The current study also underscores the uniqueness of the beaver acarofauna and reveals previously unknown biological features within the Demodecidae, including the transfer of eggs and probably larvae.

## 1. Introduction

The Eurasian beaver *Castor fiber* Linnaeus, 1758, is renowned as the largest European rodent. It belongs to the only existing genus of the Castoridae family, comprising two species with similar biology and ecology but with historically different geographic ranges: *C. fiber* is found in Eurasia while the American beaver *C. canadensis* Kuhl, 1820, is associated with North America [1,2]. The American beaver was introduced to Europe in the 20th century, but interbreeding of both species is ruled out by karyotypic differences: *C. fiber* possesses 48 pairs of chromosomes, while *C. canadensis* has 40 pairs [3]. This undoubtedly constitutes a limitation in the exchange of parasitofauna with other host species, especially parasites transmitted through direct contact [4].

Unlike many other animals, the beaver is also characterized by an amphibious lifestyle that allows it to adapt its environment to its needs [5,6,7]. Due to both this unique lifestyle and their evolutionary history and lineage, beavers should be characterized by a distinct, sometimes unique parasitofauna [4]. This especially applies to parasitic arthropods, which are often transferred directly between hosts during inter-individual contact; they are also rarer in aquatic and amphibious mammals, due to the lifestyle of ectoparasites typical of terrestrial mammals [8].

The unique nature and specificity of the arthropods constituting the parasite fauna of beavers is undoubtedly best illustrated by the mites from the genus *Schizocarpus* (Astigmata: Chirodiscidae), recorded only in the genus *Castor*, or the beetle *Platypsyllus castoris* Ritsema, 1869 (Coleoptera: Leiodidae) [8,9,10,11,12,13,14,15,16,17]. In contrast, due to their ability to inhabit skin structures and various tissues of the host, the skin mites of the Demodecidae are not restricted by their living environment [8]. Nevertheless, they are also undoubtedly characterized by host specificity: individual mammals are known to be associated with specific species inhabiting various microhabitats within the host [18].

The Demodecidae acarofauna of mammals shows both certain universal and unique elements characteristic of a given host group. In some hosts, unique species with interesting structural modifications and adaptations have been described, such as *Glossicodex musculi* Izdebska *et* Rolbiecki, 2016, from the tongue of the house mouse *Mus musculus* Linnaeus, 1758; or *Miridex putorii* Izdebska, Rolbiecki *et* Rehbein, 2022, from the vibrissae of the European polecat *Mustela putorius* Linnaeus, 1758; or *Demodex desmodi* Desch, 1994, from the vampire bat *Desmodus rotundus* (E. Geoffroy, 1810) [19,20,21]. So far, the greatest biodiversity of these mites has been described in rodents from the murids, with distinct species inhabiting the hairy skin of the entire body in individual hosts. However, many other species exist whose microhabitats are limited to the eye area, auditory canals, non-hairy skin (e.g., lips), oral cavity (tongue, gums), nose, or the region of the vibrissae [18].

Studies on the European beaver represent an interesting addition to the state of knowledge about the occurrence of demodecid mites in rodents. It is unknown whether the demodecid mite fauna is unique to this host, as is the case for other parasitic arthropods, or whether, as in many other mammals, a dominant species is associated with the hairy skin of the entire body, and rarer, co-occurring species with separate, spatially restricted microhabitats. So far, only *Demodex castoris* Izdebska, Fryderyk *et* Rolbiecki, 2016, has been recorded in the genus *Castor*; this specimen was retrieved from the skin around the nose on a Eurasian beaver, with a low level of infection [8]. However, as the entire skin of this host has not been comprehensively studied, the aim of the current study was to confirm the presence of other co-occurring species across the full topography of *D. castoris*. The analysis resulted in the discovery of a new species: *Demodex ovaportans* sp. nov.

## 2. Materials and Methods

Four Eurasian beavers from Poland (Warmian–Masurian Voivodeship, Lake Smolak, 53°43′26″ N 21°36′09″ E, Onufryjewo, 53°41′29″ N 21°36′14″ E, and Popielno, 53°45′05″ N 21°37′34″ E), collected in April 2011, were examined for demodecid mites.

Demodecidae were isolated using the digestion method developed for the detection of mammalian skin mites [22], with modifications made to suit the examined host. Skin fragments of 1 cm^2^ were taken from various areas of the body, including the head (around the eyes, nose, area of vibrissae, lips, chin, cheeks, ear pinnae, and vertex), neck, abdomen, back, limbs, tail, and the genital–anal area. Skin samples were preserved in 70% ethanol and subjected to digestion in a 10% potassium hydroxide solution. The digestion material was decanted (examination of 1 cm^2^ of the skin equal to the analysis of approximately 100 wet preparations), mounted, and examined under phase-contrast microscopy (Nikon Eclipse 50i, Tokyo, Japan). The mites were placed in a polyvinyl-lactophenol solution and measured as follows: total body length = length of gnathosoma, podosoma, and opisthosoma; gnathosomal width (at base); and podosomal and opisthosomal width (maximum width). All measurements are given in micrometers.

The specimen depositories are cited using the following abbreviation: UGDIZP, University of Gdańsk, Department of Invertebrate Zoology and Parasitology, Gdańsk, Poland [23].

The description of the species adopted the nomenclature commonly used for the family Demodecidae [24] and was completed with the nomenclature proposed by Bochkov [25] for the superfamily Cheyletoidea (Acariformes: Prostigmata) and by Izdebska and Rolbiecki [19]. The scientific and common names of the hosts follow Wilson and Reeder [1] and the Integrated Taxonomic Information System [26].

The prevalence (percentage of hosts infected) and density (number of parasites per unit area) were calculated to determine the level of host infection [27].

All applicable institutional, national, and international guidelines for the care and use of animals were followed. Ethical permission for the research was granted by the 3rd Local Ethical Committee on Animal Testing in Warsaw, Poland (Resolution No. 11/2010, 28 January 2010) and by the Regional Directors for Environmental Protection in Olsztyn, Poland (Resolution No. RDOŚ-28-OOP-6631-0007-638/09/10/pj, 25 January 2010).

## 3. Results

### 3.1. Systematics (Table 1, Figure 1 and Figure 2)

*Demodex ovaportans* sp. nov. Izdebska, Rolbiecki, Dzido *et* Fryderyk, 2025

Female (n = 68 paratypes and 1 holotype). Body elongated, slender, conical; 281 µm (243–318 µm) long and 50 µm (43–60 µm) wide (holotype, 288 × 49 µm). Gnathosoma poorly separated from podosoma; oval, with length smaller than width at base; on dorsal side, in middle part of basal (coxal) segment, pair of small club-shaped supracoxal spines (setae *elc.p*) present, ca. 1.5–2.0 µm long (holotype, 2.0 µm), directed obliquely, anterolaterally (outward, upward). Palps 3-segmented, terminating in three unbifurcated spines on tibio–tarsus. On ventral surface of gnathosoma, horseshoe-shaped pharyngeal bulb with pair of very small subgnathosomal setae (setae *n*) situated clearly above anterior on both sides. Podosoma cylindrical; four pairs of short legs, with coxa integrated into ventral idiosomal wall and five free, overlapping segments (trochanter–tarsus); two strongly bifurcated claws, ca. 6.0 µm long (holotype, 6.0 µm), with sharp spur and triangular bulge on each tarsus. Epimeral plates (coxal fields) I–III distinctly sclerotized, pair IV weakly sclerotized; pairs I and IV trapezoidal, pairs II and III rectangular. On dorsal side of podosoma, podosomal shield present, reaching level of legs III. Opisthosoma elongated, conical pointed at end, constitutes 69% (65–74%) of body length (holotype, 71%). Whole opisthosoma delicate annulated. Opisthosomal organ present; tubular in shape, elongated, ca. 15 µm in length; located in posterior part of opisthosoma. Vulva 11 µm (9–16 µm) long (holotype, 11), located distinctly (ca. 5 µm) below posterior edges of epimeral plates IV.

Male (n = 6 paratypes). Cylindrical, more slender than female, 179 µm (145–218 µm) long, 41 µm (38–46 µm) wide. Gnathosoma shape similar to female. Pharyngeal bulb and morphological details of gnathosoma similar to those of female but usually more delicate (slightly smaller). Shape of podosoma and legs also similar to those of female, but epimeral plates separated medially. On dorsal side of podosoma, podosomal shield present, reaching level of legs III. Opisthosoma narrow, cylindrical, slightly tapering towards end, pointed at end, constitutes 59% (52–67%) of body length. Whole opisthosoma distinctly annulated; annuli relatively wide at ca. 3–4 µm. Opisthosomal organ not visible. Aedeagus stocky, 15 µm (13–17 µm) long, on dorsal surface, located between epimeral plates I and II. Genital opening located on dorsal surface at level of medial part of epimeral plate I.

Egg (n = 18): Delicate, oval, non-operculate, 40 (35–43) long and 31 (24–33) wide.

Immature stages: Mainly adults and eggs were found, as well as two larvae, attached, like the eggs, to the dorsal side of the female podosoma (podosoma shield); it was not possible to analyze its features.

Material deposition: Female holotype (reg. no. UGDIZPMCfDDo20f), 68 female paratypes reg. no. UGDIZPMCfDDo01f–19f, UGDIZPMCfDDo21f–69f), six male paratypes (reg. no. UGDIZPMCfDDo01m–06m); hairless skin of the head, mainly around the mouth (lips, chin, cheek, eyelid, and nose); host *Castor fiber* (reg. no. MCCf01–04); Lake Smolak, Onufryjewo, Popielno, Poland; April 2011; parasites coll. L. Rolbiecki, J.N. Izdebska, and J. Dzido; host/skin coll. S. Fryderyk; the whole-type material (mounted microscope slides with the demodecid mites) deposited within the framework of the Collection of Extant Invertebrates in Department of Invertebrate Zoology and Parasitology, University of Gdańsk, Poland.

Etymology: The specific epithet *ovaportans* refers to the biology of the species and denotes an egg-bearing demodecid mite.

**Figure 1:**
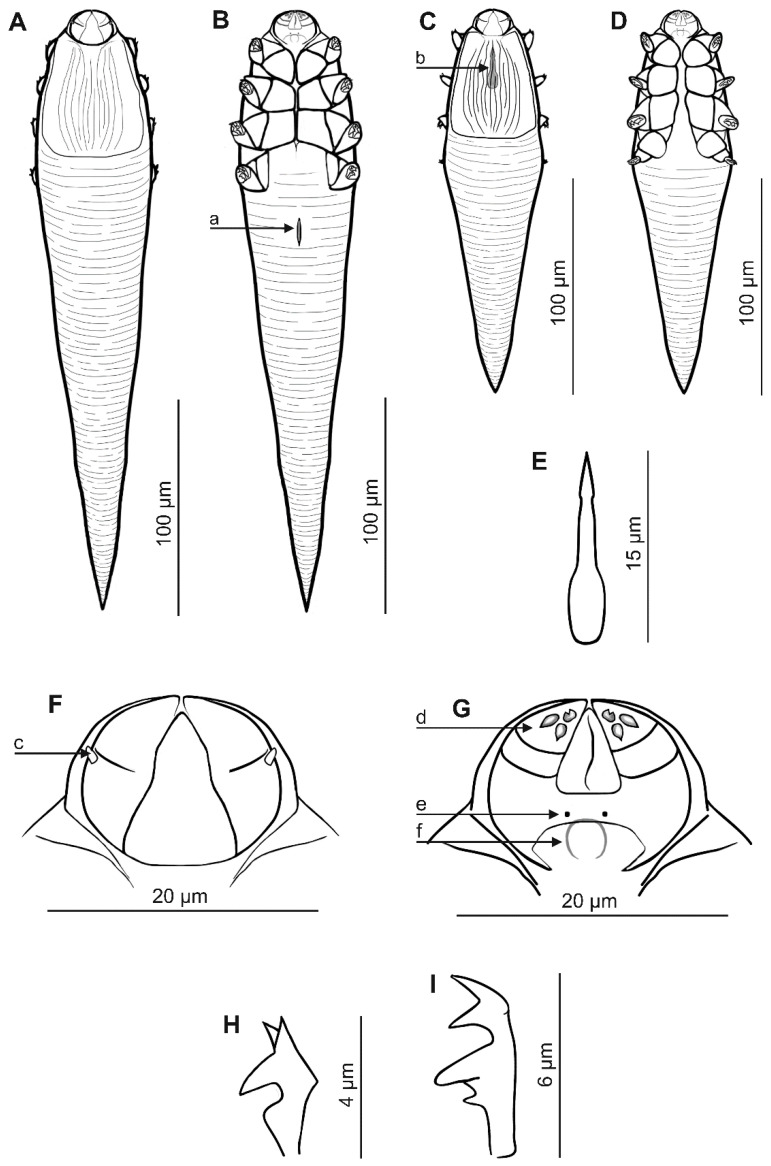
*Demodex ovaportans* sp. nov.: female, dorsal view (**A**), female, ventral view (**B**), male, dorsal view (**C**), male, ventral view (**D**), aedeagus (**E**), gnathosoma, female, dorsal view (**F**), gnathosoma, female, ventral view (**G**), and claw on the leg (**I**); a: vulva, b: aedeagus, c: supracoxal spine (seta *elc.p*), d: spines on palps, e: subgnathosomal seta (seta *n*), f: pharyngeal bulb, and *Demodex castoris*: claw on the leg (**H**).

**Figure 2:**
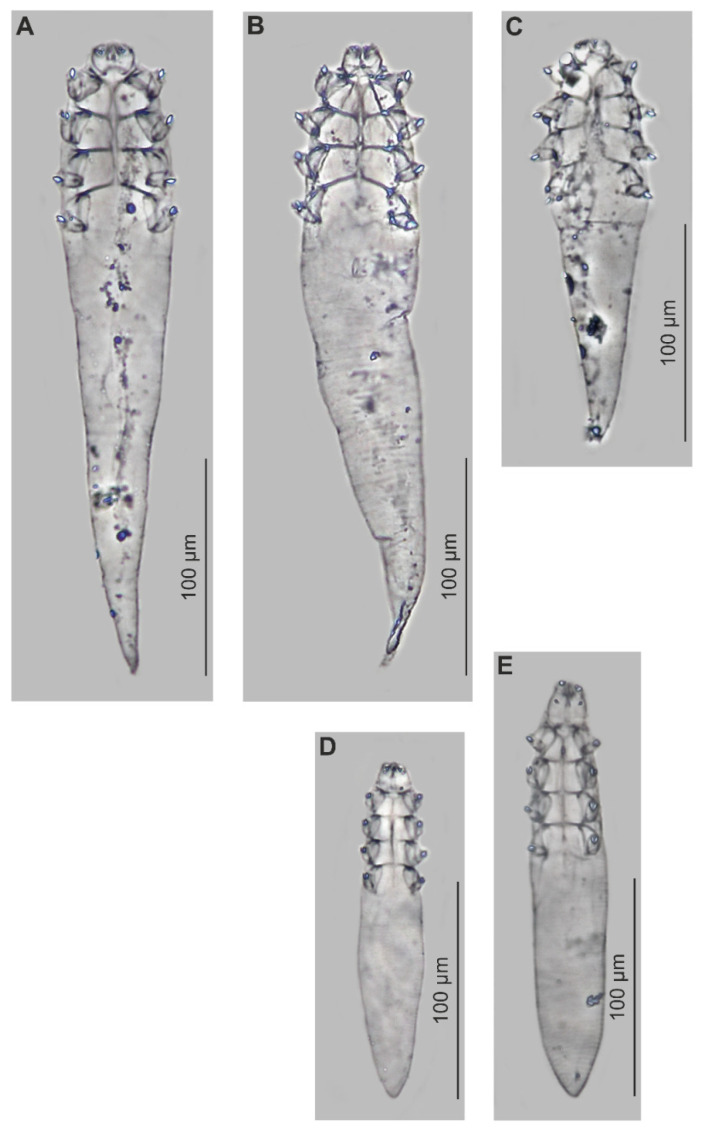
*Demodex ovaportans*. sp. nov.: female, various morphotypes (**A,B**), male (**C**) and *Demodex castoris*: male (**D**), and female (**E**).

Differential diagnosis: *Demodex ovaportans* sp. nov., compared to the *D. castoris* previously described from the same host ([8] and authors’ unpublished data), shows clear differences in characteristics important for the taxonomy of Demodecidae (Table 1, Figure 1 and Figure 2), as well as in the body shape and proportions. *Demodex ovaportans* sp. nov. is larger; the females in particular are distinctly longer and wider than those of *D. castoris*. In addition, *D. ovaportans* sp. nov. females also have a relatively longer opisthosoma. The gnathosoma of. *D. ovaportans* sp. nov. is oval with a length smaller than the width at the base, while that in *D. castoris* is trapezoidal, clearly separated from the podosoma, with a length similar to or greater than the width at the base. Other differences between these species also concern the important structures of the gnathosoma. The supracoxal spines in *D. ovaportans* sp. nov. are club-shaped, directed obliquely and anterolaterally; in *D. castoris*, they are conical and directed medially. Both species present three spines on the terminal segments of the palpi; however, they are similar in size in *D. ovaportans* sp. nov, while one is smaller and two are larger in *D. castoris*. The subgnathosomal setae in *D. ovaportans* sp. nov. are located on both sides of the pharyngeal bulb but clearly above its anterior edge; in *D. castoris*, they are also on both sides of the pharyngeal bulb, but at the level of its anterior edge. The differences also concern the structure of the leg elements: the epimeral plates are separated medially in *D. ovaportans* sp. nov. males (they do not connect in the middle part of the podosoma) but connect medially in *D. castoris* males. In addition, the legs differ: the claws are larger (6 µm) and more massive in *D. ovaportans* sp. nov., while they are smaller (4 µm) in *D. castoris*. The IV pair of epimeral plates in *D. ovaportans* sp. nov. females is weakly sclerotized, and the vulva is located clearly below its posterior edge (ca. 5 µm). Moreover, the IV pair of epimeral plates is well sclerotized in *D. castoris* females, and the vulva is located in a triangular incision between these plates. Furthermore, the aedeagus of the male *D. ovaportans* sp. nov. is relatively short (13–17 µm), located at the level of epimeral plates I and II (genital orifice at the level of the medial part of epimeral plate I), while it is longer in *D. castoris* (20–26 µm) and located at the level of epimeral plates II and III (genital orifice at the level of the posterior part of epimeral plate I). The distinctiveness of the species is also confirmed by parasitological data regarding location preferences: *D. ovaportans* sp. nov. was found in the hairless skin of the head, mainly around the mouth, while *D. castoris* is associated with various regions of the hairy skin of the body.

### 3.2. Infestation and Biological Data

**Figure 3:**
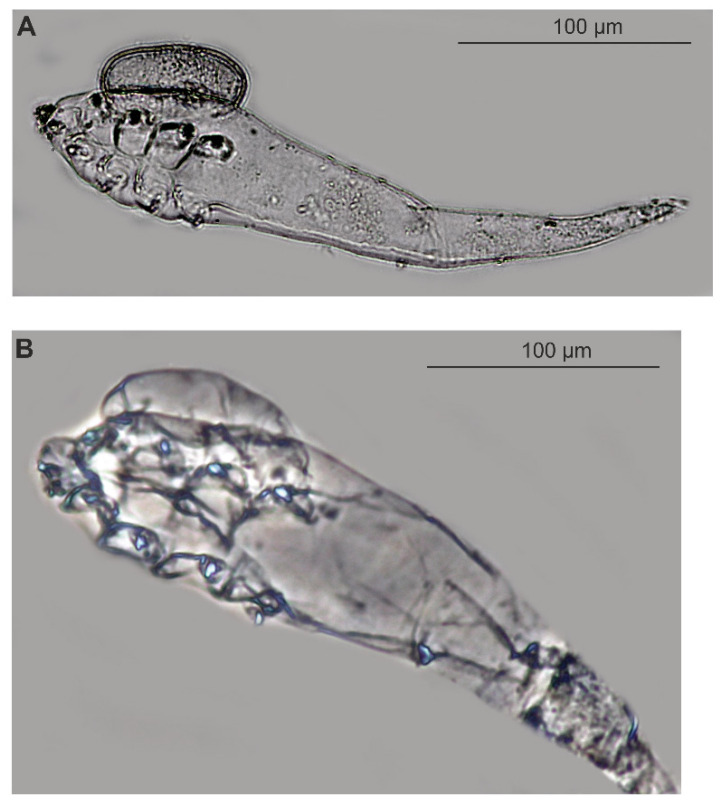
*Demodex ovaportans* sp. nov.: female with egg (**A**), female with larva (**B**).

*Demodex ovaportans* sp. nov. was found in 100% of the examined Eurasian beavers, with a mean density of 9.4 individuals. The mite population collected in April mainly consisted of adults (69 females, six males), with 18 females bearing one egg each and 2 females bearing one larva (Figure 3).

**Figure 4:**
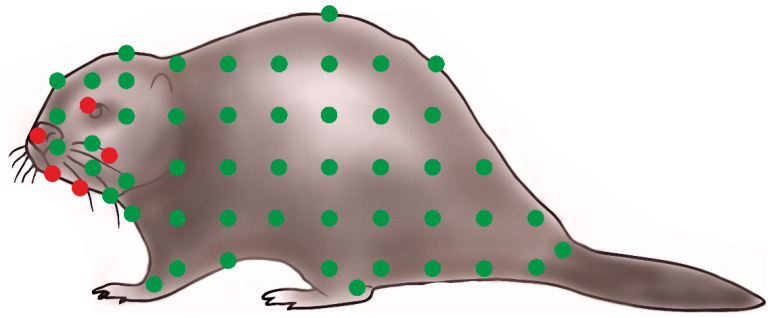
Topography preferences of demodecid mites on the Eurasian beaver: 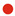 *Demodex ovaportans* sp. nov., 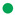 *Demodex castoris*.

The eggs were located in the region of the dorsal shield on the podosoma. In two females, larvae were found attached in the same place. *Demodex ovaportans* sp. nov. were found in the hairless skin of the head, mainly around the mouth (lips: 66 specimens/density 22.0, chin: 5 specimens/density 5.0, cheek: 1 specimen/density 1.0, eyelid: 1 specimen/ density 1.0, and nose: 2 specimens/density 1.0) (Figure 4).

Besides *D. ovaportans* sp. nov., *D. castoris* were also observed, with a prevalence of 100%. The latter species was associated with the entire hairy skin of the body (head, abdomen, back, thorax, and limbs) (Figure 4).

The infestation did not cause skin lesions in the examined hosts.

## 4. Discussion

Research has found the mites of the Demodecidae to demonstrate a similar model of colonization in mammals: the microhabitats of the host skin are colonized by different species to make optimal use of the body as a living environment. In all cases, the dominant parasite species was associated with the entire hairy skin of the body, while the subsequent, co-occurring species were associated with limited microhabitats. As a rule, the latter were rarer and showed lower infestation rates [19,28,29,30,31,32].

In the Eurasian beaver, only one species of Demodecidae, *D. castoris*, had previously been found, with the specimen retrieved from a location limited to the area around the nose. Current comprehensive topography studies have found this to be the dominant species in the beaver, being found throughout the entire hairy skin of the body and in potentially very large numbers, with areas of high density. Despite this, its presence did not appear to have caused any skin changes in the examined beavers, which may indicate that it is a specific species, well adapted, and therefore tolerated. The distribution of *D. castoris* in the skin was not even: the largest number of specimens were found in the head region (hairy skin of the nose region, vibrissae, chin, and eye area), abdomen, and limbs, but they were also observed in other regions of the trunk and the caudal regions (Figure 4).

In addition, our observations have identified a new species, *D. ovaportans* sp. nov., with a localization limited to sparsely haired and hairless skin, mainly the periorbital regions. The new species is characterized by clear morphological differences to *D. castoris*, particularly those playing key roles in the taxonomy of Demodecidae [19]. *Demodex castoris* itself was also found to inhabit other regions of the beaver, and although the two species sometimes demonstrated adjacent areas of occurrence, no individuals with mosaic features were found, which confirms their species distinction.

In the population structure of *D. ovaportans* sp. nov., females dominated over males (12:1), and no free/active juvenile stages (larvae, nymphs) were observed, which may have resulted from the stage of population development. An interesting phenomenon was also observed, where single eggs were attached to the dorsal shield of females (Figure 3), always in the same location. So far, Demodecidae eggs have been observed only inside the females, i.e., during development and preparation for laying, or they occurred freely in the studied sample, not attached to adults. The regularity of this phenomenon may suggest that it is not accidental. Confirmation may be provided by finding a developed larval stage in one female in the same location. In mites, reproductive strategies related to caring for eggs or offspring are relatively rare and usually have adaptive significance. Care for laid eggs has been observed, for example, in *Cheyletus eruditus* (Schrank, 1781) (Acariformes: Cheyletidae), where the female creates a deposit of eggs, then sits on it until the larvae hatch [33,34]. It is difficult to clearly determine the significance of the attachment of eggs to females of *D. ovaportans* sp. nov. as it was not possible to observe the full development cycle. Perhaps the attachment of eggs to the female’s body is a variant of care not yet observed in Demodecidae mites.

## 5. Conclusions

To summarize, our findings are in line with the model of Demodecidae parasitism noted in many other hosts, i.e., where the host body is inhabited by co-occurring species adapted to different skin microhabitats or other structures or organs. Here, the dominant species, associated with the entire hairy skin of the body, was *D. castoris*, previously known from one record and one location/microhabitat. A co-occurring species, *D. ovaportans* sp. nov., associated with the hairless or sparsely haired areas of the skin, was also discovered and described; some females of the latter were also found to carry attached eggs, which is the first observation of such an egg–female association in demodecid mites and may represent a form of care for the offspring.

## Figures and Tables

**Table 1:** Characteristic features of *Demodex* species collected from *Castor fiber*.

Feature/Species	*Demodex ovaportans* sp. nov.		*Demodex castoris*	
Source	Present Study		Izdebska et al. [8] and Authors’ Unpublished Data	
Sex Sample Size	Males (n = 6)	Females (n = 69)	Males (n = 16)	Females (n = 27)
Length of gnathosoma	13 (11–13) ± 1	14 (13–17) ± 1	18 (15–23) ± 2	19 (16–22) ± 2
Width of gnathosoma (at base)	19 (18–20) ± 1	22 (20–25) ± 1	18 (14–24) ± 2	18 (14–22) ± 2
Length of podosoma	60 (55–65) ± 3	72 (63–80) ± 3	49 (45–63) ± 4	60 (50–68) ± 4
Width of podosoma	41 (38–46) ± 3	50 (43–60) ± 3	28 (21–33) ± 3	27 (22–35) ± 3
Length of opisthosoma	107 (75–147) ± 25	195 (158–232) ± 16	85 (70–100) ± 8	106 (90–127) ± 9
Width of opisthosoma	36 (33–39) ± 3	46 (28–53) ± 4	26 (22–33) ± 3	32 (24–38) ± 3
Aedeagus length	15 (13–17) ± 1	-	21 (20–26) ± 2	-
Vulva length	-	11 (9–16) ± 1	-	11 (9–16) ± 2
Body total length	179 (145–218) ± 24	281 (243–318) ± 17	152 (134–168) ± 10	185 (164–209) ± 11
Body length to width ratio	4.4 (3.8–5.5) ± 0.7	5.6 (4.8–6.8) ± 0.4	5.5 (4.7–7.3) ± 0.8	5.8 (4.5–7.4) ± 1
Opisthosoma length to body length ratio (%)	59 (52–67) ± 6	69 (65–74) ± 2	56 (49–60) ± 3	57 (52–61) ± 0.02

## Data Availability

The data presented in this study are available on request from the corresponding author.
